# Primary cryoablation for Gleason 8, 9, or 10 localized prostate cancer: Biochemical and local control outcomes from the Cryo OnLine database registry

**DOI:** 10.4103/0970-1591.44254

**Published:** 2008

**Authors:** J. Stephen Jones, John C. Rewcastle

**Affiliations:** Glickman Urological Institute, Cleveland Clinic Foundation, Cleveland, OH, USA; 1 Department of Radiology, University of Calgary, Calgary, AB, Canada

**Keywords:** Cryotherapy, prostate cancer, minimally invasive

## Abstract

**Introduction and Objective:**

The increased use of cryoablation as an initial treatment for localized high-grade prostate cancer has been due to many factors including reports that cell kill from exposure to cryogenic temperatures is independent of cellular dedifferentiation and Gleason score. The objective of this study is to report the outcomes of primary cryoablation when used to treat Gleason 8, 9, or 10 localized prostate cancer at a large number of centers.

**Materials and Methods:**

Data from 1608 patients who underwent primary cryoablation at 27 centers were collected using the Cryo OnLine Database (COLD) registry. This analysis considers only the 77 patients who had a Gleason score of at least 8 and a minimum of 24 months of follow-up. Biochemical failure was defined according to both the original ASTRO definition (three rises) and the 2006 updated ASTRO (Phoenix) definition of nadir + 2. Biopsy was performed at the physician's discretion, but most commonly if a patient had a rising or suspicious prostate specific antigen (PSA).

**Results:**

The average age at treatment was 69.6 ± 8.2 years. Pretreatment PSA was 16.2 ± 17.9 ng/ml and the average Gleason was 8.5 ± 0.6. Patients were followed for 39.0 ± 18.8 months (range: 24-120 months) and 5-year follow-up was available for 12 patients. Eight-seven percent of the patients achieved a PSA nadir < 0.4 ng/ml. Five-year actuarial biochemical survivals was 64.4 ± 6.0% and 44.6 ± 8.0% for the ASTRO and Phoenix definitions, respectively. A total of 47 underwent posttreatment biopsy. Of these, 12 showed evidence of disease resulting in a positive biopsy rate for those who underwent biopsy of 25.5%. This yields a positive biopsy rate of the entire population of 15.6% (12/77).

**Conclusions:**

Cryoablation, as a primary treatment for high-grade Gleason prostate cancer practiced over a wide spectrum of users provides definable biochemical and local control for a hard to manage patient population with aggressive disease.

## INTRODUCTION

Despite the well-recognized stage migration that has led to an improvement in prostate cancer outcomes since the era of prostate specific antigen (PSA)-based prostate cancer screening began, patients with high-grade prostate cancer remain at significant risk of morbidity and mortality.[1] Patients with Gleason score >8 cancer are often treated with radical prostatectomy or radiotherapy combined with adjuvant androgen blockade. Some patients have confounding factors limiting their ability to undergo surgery, including age, anesthetic risks, or simply refusal of surgical intervention. External beam radiotherapy in combination with 6-24 months' hormonal ablation offers a nonsurgical alternative, and brachytherapy or the combination of external beam radiotherapy with brachytherapy is used for some high-risk patients as well. These options carry substantial risk of complications, including erectile dysfunction, incontinence, or damage to the surrounding structures that continue to limit their acceptance for some patients. Moreover, the efficacy of any modality decreases in patients with high-grade disease.[1]

Cryosurgical ablation has been used in many centers as a minimally invasive alternative to surgery and radiation that has the potential to eradicate prostate cancer irrespective of tumor grade. Unlike radiotherapy, cryoablation appears to reproducibly kill all cells frozen to lethal temperatures.[2] This has led to the suggestion that it may be preferentially useful for patients with high-grade disease.[3]

This study reports a relatively large group of patients diagnosed with Gleason 8, 9, or 10 prostate cancer who underwent cryoablation as their primary therapy. Patient data were collected with the Cryo OnLine Database (COLD) registry.

## MATERIALS AND METHODS

The COLD registry is a secure web-based registry consisting of case report forms designed to collect relevant pre- and post-treatment information for patients undergoing prostate cryoablation. A central institutional review board's approval covers the collection and analysis of deidentified data, additional IRB approval for individual centers has been obtained when required by institutional policy. Investigators who have entered data into the COLD registry are listed in the acknowledgments.

Only patients with Gleason score 8 or greater having minimum 24 months follow-up were included in this analysis. Patients undergoing cryotherapy following failure of prior definitive radiotherapy or brachytherapy, and those that had undergone focal, nerve sparing, or nerve warming treatment were excluded. A total of 77 patients from 27 centers were identified meeting the criteria.

Biochemical failure was defined according to both the original ASTRO definition of three consecutive rises following nadir (referred to as “ASTRO”), and the second ASTRO definition of nadir + 2 (referred to as the “Phoenix” definition). Biopsy was performed at the physician's discretion, but most commonly if a patient had a rising or suspicious PSA.

Kaplan-Meier analysis was used to generate curves showing the probability biochemical failure as a function of time. Statistical analysis was performed with a commercially available statistical software package (MedCalc, Mariakerke, Belgium).

## RESULTS

**Figure 1:**
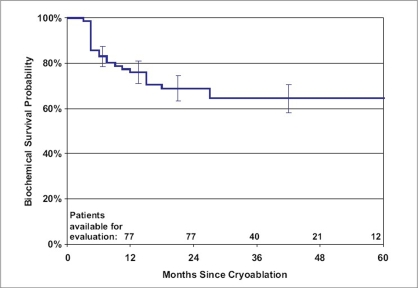
Kaplan-Meier curve of the cumulative biochemical disease free survival probability using (a) the ASTRO definition and (b) the Phoenix definition

Data were collected from 27 investigators. The average age of the 77 patients who met the inclusions criteria was 69.6 ± 8.2 years. Pretreatment PSA was 16.2 ± 17.9 ng/ml and the average Gleason was 8.5 ± 0.6. Patients were followed for 39.0 ± 18.8 months (range: 24-120 months) and 12 patients had a follow-up of at least 60 months. Posttreatment, 87% of the patients achieved a PSA nadir < 0.4 ng/ml. The 5-year actuarial biochemical survivals are 64.4 ± 6.0% and 44.6 ± 8.0% for the ASTRO and Phoenix definitions, respectively [Figure 1]. After treatment, 47 underwent biopsy. Of these, 12 showed evidence of disease. The positive biopsy rate, for those who underwent posttreatment biopsy was 25.5% and the rate for the entire population was 15.6% (12/77). No fistulas were identified in any of the patients.

## DISCUSSION

The COLD registry project is designed to address the relative paucity of published data regarding cryotherapy for prostate cancer. It is notable, however, that opinions regarding the quality and quantity of data published on radical prostatectomy, external beam radiation, and active surveillance are perhaps overstated. For example, the Kaplan-Meier 5-year biochemical disease free survival data for high-risk prostate cancer managed with external beam radiotherapy as shown in Campbell's Urology textbook is based upon a surprisingly low six patients with at least 60 months follow-up. Similarly, the present series reports only 12 patients with results at 5 years. We believe that both datasets are inadequate to make solid declarations of long-term efficacy. That being said, the authors do feel it is important to realize that the data supporting any form of treatment for the high-risk patient is lacking[1] and that the concept that radiation therapy is clearly established as a more efficacious treatment for high-risk disease must be scrutinized.

Emerging technologies are often reserved in initial series to low-risk patients in whom failure to control disease carries less risk of metastasis and death. For example, most initial brachytherapy series are heavily weighted toward patients with low-risk disease.[4] Moreover, even recent radical prostatectomy and radiation series include more low risk than high-risk patients, but this is at least partially due to high-risk disease being less common in screened populations.[5,6] In contrast, early pioneers of cryotherapy advocated the position that if lethal cold temperatures are achieved, uniform necrosis results and cell death occurs irrespective of cellular differentiation or Gleason score.[2] In addition, publications suggesting its potential to treat locally advanced disease has led to the concept of freezing beyond the prostatic capsule to eliminate extraprostatic disease that would have resulted in a positive surgical margin had radical prostatectomy been performed.[7] As a result, cryoablation is often utilized for the treatment of high-risk localized prostate cancer. Several publications of intermediate-term results have suggested that the efficacy of the procedure appears to be equivalent to radiation and surgery for low-risk prostate cancer and potentially more efficacious for moderate- and high-risk prostate cancer.[8–11] A summary of the efficacies reported in the four publications reporting 5 year outcomes exclusively for primary cryoablation is presented in Table 1.

**Table 1:** Biochemical disease free survival at 5 years following cryoablation: Reports in the literature

Study	n	Failure definition	bDFS (%) Risk group		
			
			Low	Moderate	High
Long *et al.*[9]	975	PSA < 0.5	60	61	45
		PSA < 1.0	76	71	36
Bahn *et al.*[11]	590	PSA < 0.5	64	70	65
		PSA < 1.0	86	81	76
		ASTRO	92	89	89
Donnelly *et al.*[10]	76	PSA < 0.3	-	77	48
		PSA < 1.0	-	89	76
Prepelica *et al*.[12]	65	ASTRO	-	-	82

As of yet there is no universally agreed upon definition of biochemical failure following cryotherapy, but it should be noted that consensus definitions also remain relatively elusive following radiation or surgery. Radical prostatectomy completely removes the prostate and should theoretically yield an undetectable PSA. However, some residual PSA remains detectable in many patients due to a small number of remaining benign glands. The American Urological Association (AUA) Guidelines Panel recommended in 2007 that the definition of biochemical success does not require an undetectable PSA, and that failure should be a PSA > 0.2 ng/ml confirmed by a second PSA reading > 0.2.[12] Further, the AUA panel found 166 different published definitions of biochemical failure, including 99 for patients undergoing radiation failure. In 1998 the first ASTRO definition of three consecutive rises, backdated to the midpoint of the first rise, was agreed upon by a consensus panel and became standard practice. It is the ASTRO definition that has been used most commonly following cryoablation. This is based on the similarity of cryoablation and radiation therapy in that both modalities result in some residual prostatic tissue and low but measurable PSA levels in most patients. The second ASTRO definition, dubbed the “Phoenix” definition, remains somewhat controversial in the eyes of many urologists. Although it was specified that it was not intended for use with patients undergoing cryotherapy, we have included our results using this as an additional definition solely for comparative purposes.[13]

Early cryotherapy experience made it clear that specific thresholds may be meaningless due to PSA production by residual tissue surrounding the urethra or in a benign median lobe, and the fact that a PSA of 0.4 is expected when 1 g of prostate tissue has been preserved in men free of prostate cancer.[14] It is intended that the data set collected with the COLD registry will be used to create a scientifically based definition of biochemical failure that is specific to primary prostate cancer cryoablation as these data accumulate and mature.

The use of negative biopsies as a surrogate for disease control is also controversial. Regardless of the intervention, sampling error underestimates disease when using biopsy as a surrogate. This is especially likely when small volume disease is present, as evidenced by the 61% of men known to have prostate cancer that have a negative repeat sextant biopsy when evaluated on an active surveillance protocol.[15] Seventeen percent of patients with known prostate cancer on an active surveillance protocol have a negative biopsy even when saturation biopsy is performed.[16] Some authors suggest that histological evidence of malignancy identified on biopsy should not be regarded as cancer in some postradiation settings.[17] In contrast, following cryoablation, histological results tend to fall into one of three definitive categories: fibrous tissue (scar) indicating tumor eradication, benign prostate tissue, or prostate cancer.[18] When cryotherapy was first investigated most patients underwent posttreatment biopsy to confirm local control. However, due to high-negative biopsy rates (82-98%)[8,9] most practitioners now utilize biopsy only to investigate suspicious PSA values or patterns.[19] It is not possible to know what the positive biopsy rate would be for those not determined to warrant biopsy by the treating physicians, but previous series have suggested that the likelihood of residual disease is low as demonstrated in the table.

The primary limitation of this series is the retrospective nature of a registry. There is the potential that patients with unfavorable features are not voluntarily reported to the registry, or that unfavorable outcomes are inaccurately reported. Our experience in dealing directly with the physician members and with internal audit is that we have found no evidence that there is any case selection, and to our knowledge all cases of the enrolling physicians are included. In addition, a wide variety of surgical techniques is possible with the large number of institutions participating. However, a primary goal of the COLD registry is to determine outcomes in “the real world” without the inherent reporting bias of single surgeon series. These data suggest that bDFS and complication rates are consistent with earlier single-center reports.

## CONCLUSION

The biochemical and local control of cryoablation for high Gleason score prostate cancer appear to be consistent both with early reports of cryoablation and with large series reporting experience with radiation and surgery. Improving data available on all treatment modalities for localized prostate cancer is mandatory for patients to make an informed decision on therapy.

## References

[1] Fletcher SG, Theodorescu D (2005). Surgery or radiation: what is the optimal management for locally advanced prostate cancer?. Can J Urol.

[2] Larson TR, Rrobertson DW, Corica A, Bostwick DG (2000). *In vivo* interstitial temperature mapping of the human prostate during cryosurgery with correlation to histopathologic outcomes. Urology.

[3] Bahn DK, Silverman P, Lee F, Badalament R, Bahn ED, Rewcastle JC (2004). In treating localized prostate cancer the efficacy of cryoablation is independent of DNA ploidy type. Technol Cancer Res Treat.

[4] Ragde H, Blasko JC, Grimm PD, Kenny GM, Sylvester JE, Hoak DC, Landin K, Cavanagh W (1997). Interstitial iodine-125 radiation without adjuvant therapy in the treatment of clinically localized prostate carcinoma. Cancer.

[5] Walsh PC (2003). Comparison of the efficacy of local therapies for localized prostate cancer in the prostate-specific antigen era: A large single-institution experience with radical prostatectomy and external-beam radiation. J Urol.

[6] D'Amico AV, Hui-Chen M, Renshaw AA, Sussman B, Roehl KA, Catalona WJ (2006). Identifying men diagnosed with clinically localized prostate cancer who are at high risk for death from prostate cancer. J Urol.

[7] Jones JS (2007). Ultrasound probe positioning to minimize the risk of rectourethral fistula during cryosurgical ablation of prostate cancer. BJU Int.

[8] Long JP, Bahn D, Lee F, Shinohara K, Chinn DO, Macaluso JN (2001). Five-year retrospective, multi-institutional pooled analysis of cancer-related outcomes after cryosurgical ablation of the prostate. Urology.

[9] Donnelly BJ, Saliken JC, Ernst DS, Ali-Ridha N, Brasher PM, Robinson JW, Rewcastle JC (2002). Prospective trial of cryosurgical ablation of the prostate: five-year results. Urology.

[10] Bahn DK, Lee F, Badalament R, Kumar A, Greski J, Chernick M (2002). Targeted cryoablation of the prostate: 7-year outcomes in the primary treatment of prostate cancer. Urology.

[11] Prepelica KL, Okeke Z, Murphy A, Katz AE (2005). Cryosurgical ablation of the prostate: high risk patient outcomes. Cancer.

[12] Cookson MS, Aus G, Burnett AL, Canby-Hagino ED, D'Amico AV, Dmochowski RR, Eton DT, Forman JD, Goldenberg SL, Hernandez J, Higano CS, Kraus SR, Moul JW, Tangen C, Thrasher JB, Thompson I (2007). Variation in the definition of biochemical recurrence in patients treated for localized prostate cancer: The American Urological Association Prostate Guidelines for Localized Prostate Cancer Update Panel report and recommendations for a standard in the reporting of surgical outcomes. J Urol.

[13] Roach M, Hanks G, Thames H, Schellhammer P, Shipley WU, Sokol GH, Sandler H (2006). Defining biochemical failure following radiotherapy with or without hormonal therapy in men with clinically localized prostate cancer: recommendations of the RTOG-ASTRO Phoenix Consensus Conference. Int J Radiat Oncol Biol Phys.

[14] Akdas A, Cevik I, Tarcan T, Turkeri L, Dalaman G, Emerk K (1997). The role of free prostate-specific antigen in the diagnosis of prostate cancer. Br J Urol.

[15] Patel MI, DeConcini DT, Lopez-Corona E, Ohori M, Wheeler T, Scardino PT (2004). An analysis of men with clinically localized prostate cancer who deferred definitive therapy. J Urol.

[16] Abouassaly R, Lane BR, Jones JS (2008). Staging saturation biopsy in patients with prostate cancer on active surveillance protocol. Urology.

[17] Petraki CD, Sfikas CP (2007). Histopathological changes induced by therapies in the benign prostate and prostate adenocarcinoma. Histol Histopathol.

[18] Donnelly BJ, Saliken JC, Ali-Ridha N, Rewcastle JC, White LJ (2001). Histological findings in the prostate two years following cryosurgical ablation. Can J Urol.

[19] Ellis DS, Manny TB, Rewcastle JC (2007). Cryoablation as primary treatment for localized prostate cancer followed by penile rehabilitation. Urology.

